# Hypoabsorption in Bariatric Surgery: Is the Benefit Worth the Risk?

**DOI:** 10.3390/medicina61030398

**Published:** 2025-02-25

**Authors:** Tala Abedalqader, Noura Jawhar, Aryan Gajjar, Ray Portela, Gerardo Perrotta, Nour El Ghazal, Simon J. Laplante, Omar M. Ghanem

**Affiliations:** Department of Surgery, Mayo Clinic, Rochester, MN 55905, USA; abedalqader.tala@mayo.edu (T.A.); jawhar.noura@mayo.edu (N.J.); aryangajjar0722@gmail.com (A.G.); rayportela10@gmail.com (R.P.); elghazal.nour@mayo.edu (N.E.G.); laplante.simon@mayo.edu (S.J.L.)

**Keywords:** hypoabsorptive bariatric procedures, malnutrition, nutritional deficiencies, one-anastomosis gastric bypass, biliopancreatic diversion with duodenal switch, single-anastomosis duodenoileostomy with sleeve

## Abstract

Metabolic and bariatric surgery has been well described in the existing literature to be an effective and safe modality for weight loss in patients with obesity. Recently, hypoabsorptive procedures such as one-anastomosis gastric bypass (OAGB), biliopancreatic diversion with duodenal switch (BPD-DS), and single-anastomosis duodenoileostomy with sleeve (SADI-S) have gained traction, particularly among patients with severe obesity. These procedures combine restrictive and hypoabsorptive mechanisms, resulting in significant and sustainable weight loss, especially in those with severe obesity and associated comorbidities. However, the risk of malnutrition and nutritional deficiency following these procedures has been a deterrent for surgeons in their adoption. This review evaluates the existing literature on the safety, efficacy, and long-term outcomes of OAGB, BPD-DS, and SADI-S. While these hypoabsorptive procedures represent highly effective options for treating obesity, the associated nutritional complications necessitate the need for long-term follow-up and supplementation and highlight the need for careful patient selection.

## 1. Introduction

Obesity has been documented as a global health issue, contributing significantly to the development of several metabolic comorbidities [1]. Due to the high societal, health-related, and financial burdens associated with obesity, pharmacologic and surgical interventions have been implemented in its management approach [2]. While the advent of anti-obesity medications has greatly impacted obesity management in the past decade, bariatric surgery continues to be the most effective and rapid management in patients with obesity, resulting in greater reduction in weight and body mass index over a shorter period of time [3,4]. In addition to their well-established safety compared to other weight-loss modalities, metabolic and bariatric surgeries (MBS) have been shown to result in significant and sustained weight loss by altering gastrointestinal anatomy and modifying the absorption of micro- and macronutrients [5,6]. Furthermore, remission of obesity-related medical conditions and improvement in patient quality of life are notable outcomes of metabolic surgery [7].

Currently, sleeve gastrectomy (SG) and Roux-en-Y bypass (RYGB) surgeries constitute the most performed bariatric surgeries [8]. One-anastomosis gastric bypass (OAGB) makes up the third most commonly performed procedure, with a reported incidence of 10% worldwide and comparable documented safety profiles and short-term outcomes with SG and RGYB [9,10,11,12]. Following the endorsement of OAGB by the American Society for Metabolic and Bariatric Surgery (ASMBS) in 2019, more OAGB surgeries have been adopted by metabolic surgeons [9]. However, there remains some reluctance in its adoption into metabolic surgery practice, which can be explained by the relatively recent emergence of this technique, lack of consensus on the optimal technique to reduce the risk of malnutrition, and the lack of comprehensive data on long-term outcomes [13,14,15,16]. Additionally, concerns over the risk of development of gastric and esophageal cancers, higher mortality rates, and inferior weight loss outcomes have been cited as factors influencing a surgeon’s decision to opt for alternative procedures [13].

Conversely, duodenal switch procedures, including biliopancreatic diversion with duodenal switch (BPD-DS) and single-anastomosis duodenal switch with sleeve (SADI-S), are less frequently performed. An international survey identified key factors in reluctance in the adoption of switch procedures, including perceptible long-term complications and lack of training [5]. Additionally, patient characteristics, including pre-operative comorbidities and preferences, play a key role in the choice of the bariatric operation performed [17].

**Figure 1:**
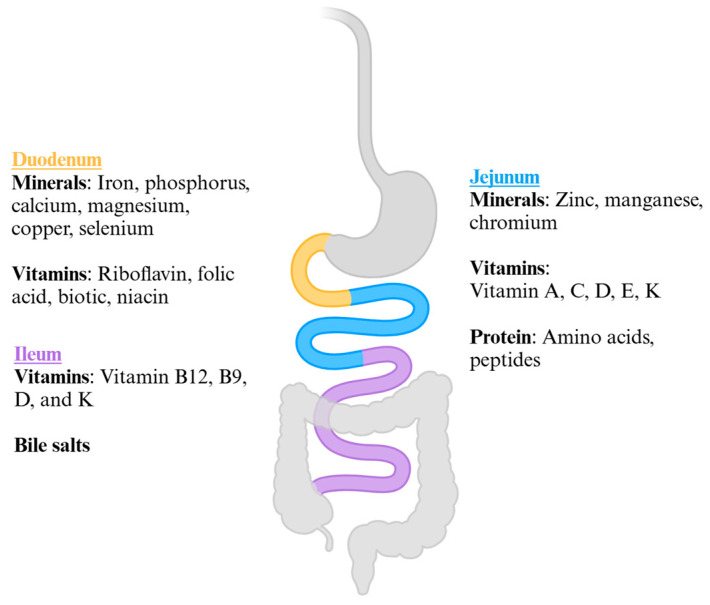
Key sites of micronutrient absorption.

Nutritional deficiencies are a main concern following bariatric surgeries, particularly duodenal switch and OAGB surgeries. Given the profound weight loss outcomes following hypoabsorptive procedures, they are especially useful in patients with severe obesity in which other bariatric surgeries and anti-obesity medications may not be as effective. Weight loss is achieved through macronutrient malabsorption, owing to a combination of reduced gastrointestinal transit time and reduced contact of ingested food with brush border enzymes. Since the bypassed duodenum and jejunum are critical sites for vitamin and mineral absorption, this also leads to impaired absorption of micro and macronutrients, leading to malnutrition and deficiencies (Figure 1) [18,19]. This comprehensive review offers critical insight into the incidence of malnutrition in BPD-DS, SADI-S, and OAGB. Additionally, we aim to address a key question: do the benefits of malabsorptive bariatric surgeries outweigh the risk of hypoabsorption and malnutrition?

## 2. Materials and Methods

**Figure 2:**
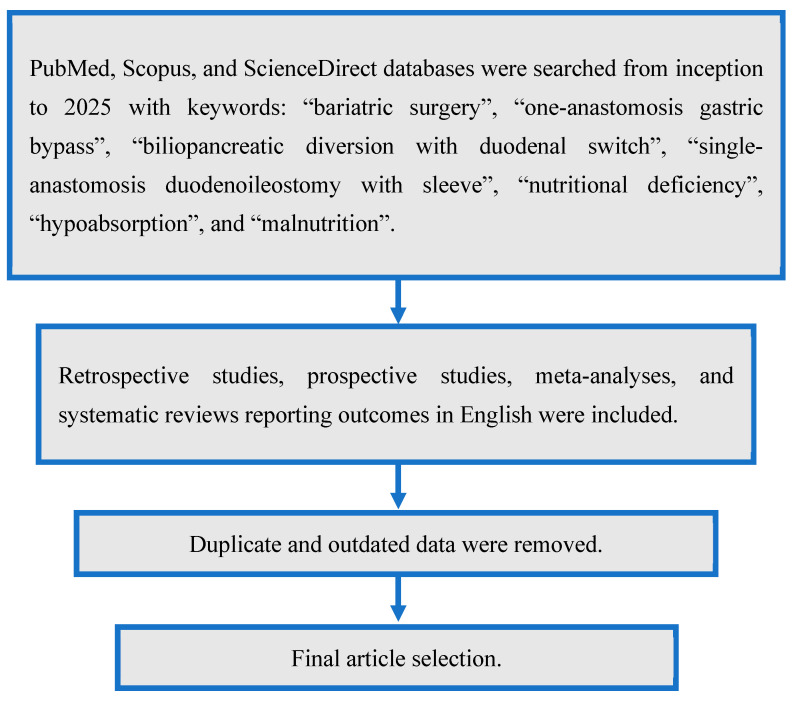
Article selection flowchart.

An extensive literature review was performed by the authors of this manuscript in three phases: identifying relevant research questions, performing the literature review, and reporting the most relevant findings. Tasks were coordinated by all the involved authors. Using PubMed, Scopus, and ScienceDirect, relevant articles were chosen from inception to 2025 according to our keywords: “bariatric surgery”, “one-anastomosis gastric bypass”, “biliopancreatic diversion with duodenal switch”, “single-anastomosis duodenoileostomy with sleeve”, “nutritional deficiency”, “hypoabsorption”, and “malnutrition”. Retrospective studies, prospective studies, meta-analyses, and systematic reviews reporting outcomes in English were included. Abstracts, non-English texts, and articles that did not include our relevant search words were excluded. Manuscripts were then screened, and duplicates were removed. The selection of articles was approved by the corresponding author, who provided his expert opinion to synthesize the information and edit the manuscript draft (Figure 2). Table 1 provides a summary of the key studies reporting nutritional outcomes after OAGB, BPD-DS, and SADI-S used to synthesize the manuscript.

## 3. One-Anastomosis Gastric Bypass (OAGB)

**Figure 3:**
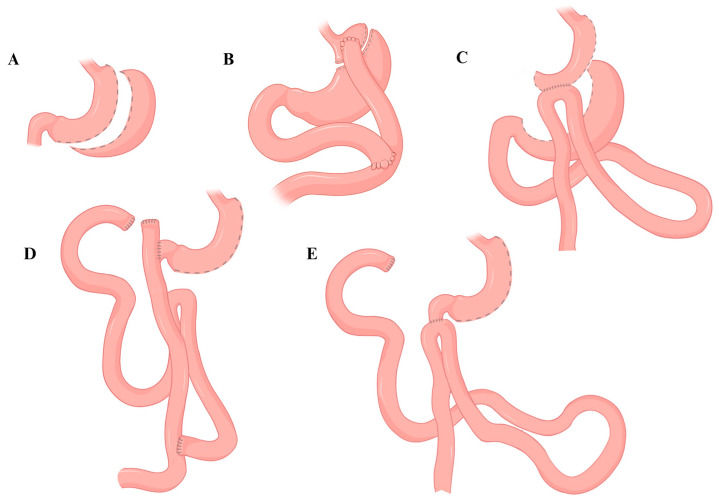
(**A**) Sleeve gastrectomy; (**B**) Roux-en-Y gastric bypass; (**C**) One-anastomosis gastric bypass; (**D**) Biliopancreatic diversion with duodenal switch; (**E**) Single-anastomosis duodenoileostomy.

A laparoscopic or minimally invasive approach is used in OAGB, and the procedure consists of two phases: gastric pouch tailoring and small bowel anastomosis. Division of the stomach is achieved using a stapler angled at a 90-degree angle to the lesser curvature, inserted through the lesser sac. The staple line is placed parallel to a 36 Fr gastric tube, beginning at the transection site and advancing towards the angle of His. The fundus is excluded, and the short gastric vessels are not divided. Subsequently, an end-to-side or side-to-side anastomosis is performed between the remaining longitudinal gastric pouch and the biliopancreatic limb, measuring 150–200 cm in length from the ligament of Treitz (Figure 3) [12,14,51].

OAGB can be performed as a primary or revisional bariatric procedure. Patient selection is dependent on multiple factors, including patient age, body mass index (BMI), associated comorbidities, and preference, as well as surgeon expertise. OAGB is typically utilized in patients with a BMI > 50 kg/m^2^ as a one-stage procedure [52,53] and can also be used in patients with a low BMI (30–35 kg/m^2^) with associated weight-related medical conditions [54]. Furthermore, OAGB has been shown to be a safe procedure in elderly patients (>65 years of age), in vegan and vegetarian patients, and in patients with large hiatal hernias needing concurrent repair, as long as there is no reflux [55].

There are several notable advantages to this operation. The literature demonstrates a significantly shorter operative period with OAGB compared to other hypoabsorptive procedures, given the single anastomosis without intestinal division. This also necessitates the closure of one instead of multiple defects, further contributing to the shorter operative period and reducing the risk of internal hernia [9,56]. Additionally, OAGB has demonstrated lower post-operative complication rates, shorter hospital length of stay, and lower reoperation rates [9].

OAGB utilizes a combination of restrictive and hypoabsorptive mechanisms to induce significant weight loss [51]. The altered gastrointestinal anatomy influences the secretion of gut hormones, such as ghrelin, glucagon-like peptide-1 (GLP-1), and insulin. Consequently, this improves satiety and insulin sensitivity, resulting in significant weight loss and resolution of diabetes [57]. Compared to RYGB and SG procedures, OAGB demonstrated similar weight loss and maintenance outcomes, at least up to 5 years post-operatively [20,56,58,59,60,61,62,63,64]. The Omega Loop Versus Roux-en-Y Gastric Bypass (YOMEGA) trial demonstrated similar percent total weight loss (%TWL) in OAGB (37.1%) and RYGB groups (35.4%) at two-year follow-up, with OAGB resulting in higher weight reduction without statistically significant differences between the two groups. In addition, a greater proportion of the OAGB group (60%) had complete remission of preoperative type 2 diabetes compared to RYGB (38%), with a proportionally greater reduction in hemoglobin A1c (HbA1c) levels (−1.2% vs. −0.6%, respectively), not reaching statistical significance [21]. Concordantly, Bhandari et al. also described superior weight loss outcomes in OAGB at five years [61]. Delko et al. reported higher % excess weight loss (%EWL) in OAGB (104.1%) than RYGB (87.9%) at 12 months, as well as better glycemic control [65]. Correspondingly, OAGB has demonstrated comparative results with RYGB and SG in the improvement of other obesity-related medical conditions [16,21,61,65,66,67].

Due to its stronger bypass component, the risk of hypoabsorption following OAGB has been recognized as a major concern and potential drawback, necessitating lifelong supplementation [68]. Malnutrition after OAGB has been reported as protein-calorie deficiency, vitamin deficiency, or anemia [15]. Because nutritional deficiencies after bariatric procedures have been extensively documented and well-recognized, nutritional supplementation has become a staple in bariatric postoperative management, with the same guidelines followed for OAGB procedures [22,23]. The majority of patients respond well to nutritional supplements without needing immediate medical or surgical (conversion/reversal) interventions [68].

Albumin is a prognostic marker in protein calorie deficit, with a concentration of less than 30 g/L characterizing malnutrition [21,24,69]. Several studies have demonstrated no significant difference in protein-calorie malnutrition in OAGB compared to RYGB [24,25,26]. The length of the afferent limb has been identified as an important contributing factor in the development of malnutrition. Although rare, severe malnutrition requiring in-hospital treatment is more common following OAGB procedures and has been shown to correlate with afferent limb length (>150 cm) [15,20,66]. Hypoalbuminemia was observed in those patients at a rate of 13.1%, which was higher than in those treated with RYGB (2%) and SG (nil) [27]. There also have been few reports of severe malnutrition requiring reversal of OAGB [26].

Anemia is reported as the most common nutritional complication following bariatric surgeries. It may be a result of deficiencies in iron, vitamin B9, or vitamin B12. The incidence of anemia is higher in OAGB and RYGB than in SG, owing to their hypoabsorptive mechanisms [26,27]. Syn et al. reported a steeper decrease in hemoglobin concentrations in patients who underwent OAGB and RYGB in the first three months compared to patients who underwent SG [28]. Other studies reported a similar trend but showed that levels remained within normal range one year post-operatively [29]. These trends indicate that prophylactic efforts against anemia should commence earlier in OAGB and RYGB patients [29].

Another anticipated complication following bariatric surgeries is vitamin and mineral deficiencies, with the most frequently reported ones including iron, ferritin, and vitamin D deficiency following OAGB [24,25,70,71,72]. It should be noted, however, that micronutrient deficiencies are prevalent in pre-operative bariatric patients [24,73,74,75,76]; thus, deficiencies diagnosed post-operatively might not be solely a consequence of the procedure performed.

Vitamin D deficiency is common pre-operatively in bariatric patients [30] but scarcely reported post-operatively, with an incidence of 4.9% [15]. Voglino et al. highlighted significant improvement in vitamin D levels post-operatively, likely due to oral supplementation and release of vitamin D from adipose tissue [30]. Hypovitaminosis B12 has been linked to multiple factors, including vitamin B12 ingestion and intrinsic factor reduction, but both B12 and folate deficiencies have been reported to be uncommon postoperative complications in OAGB patients. In fact, both vitamin levels were found to be considerably higher in the OAGB patient at 3 years post-operatively due to the supplementation regimen provided [30]. Conversely, there is a rarer occurrence of vitamin A and E deficiencies, with a mean incidence of 1.8% each over a three-year period [15,30].

Mineral deficiencies reported after bariatric surgery include iron, copper, zinc, phosphorus, and calcium [31]. In OAGB patients, there was a higher reported incidence of iron and zinc deficiencies compared to RYGB and SG patients [28]. Iron deficiency has been reported in OAGB patients at a rate of up to 10%, with comparable rates in RYGB patients but lower incidence in SG patients [70], and this can be attributed to the bypassing of the duodenum and proximal jejunum, which are the main iron absorption sites. On the other hand, zinc deficiency can be explained by protein-calorie deficiency in OAGB patients, as protein-rich foods serve as the primary source of zinc [77].

Considering the above, OAGB can lead to both micro- and macronutrient deficiencies. However, optimizing the afferent limb length can help mitigate the risk of malnutrition and associated nutritional deficiencies.

## 4. Bilio-Pancreatic Diversion with Duodenal Switch (BPD-DS)

Biliopancreatic Diversion with Duodenal Switch (BPD-DS) is another type of metabolic and bariatric surgery providing patients with an effective way in the management of obesity and associated metabolic conditions. It involves combining hypoabsorptive, restrictive, and hormonal components to improve weight loss and resolution of weight-associated diseases. The first step of BDP-DS is similar to a standard sleeve gastrectomy; the gastrocolic ligament is opened, and the greater curvature is fully mobilized from the antrum to the angle of His. Resection of the stomach is then carried out over the greater curvature over a 50F–60F bougie, about 5 cm proximal to the pylorus. Next, the ileocecal junction is identified, and the small bowel is measured at a length of 250 cm from the valve. Transection of the small bowel is performed at this point, and the alimentary limb is marked. The subsequent step involves creating an anastomosis between the duodenum and the ileum (alimentary limb). Finally, an ileo-ileal anastomosis is created 100 cm from the ileocecal valve to form a connection between the alimentary and biliary limb. Some surgeons endorse performing the distal anastomosis before the proximal anastomosis (Figure 3) [78,79,80].

The hormonal, hypoabsorptive, and restrictive processes of BPD-DS are vital in improving metabolism and inducing weight loss [8,18]. The restrictive component involves the initial sleeve gastrectomy, which, by reducing the size of the stomach, contributes to decreased caloric intake and reduced absorptive surface. The smaller absorptive surface leads to impaired digestion and absorption of macronutrients such as fats and other nutrients, further contributing to weight loss. In addition, BPD-DS has also been shown to affect glycemic control and energy homeostasis [81]. By bypassing specific parts of the small intestine, such as the duodenum and jejunum, there is an increased release of hormones such as glucagon-like peptide 1 (GLP-1), which plays a role in reducing hunger and improving insulin sensitivity [82,83], factors that aid in the remission of type 2 diabetes [83,84]. Moreover, BPD-DS can potentially alter the composition and diversity of gut microbiota, further contributing to preserved energy equilibrium, reduced inflammation, and improved glucose metabolism [85].

BPD-DS is typically reserved for patients with class IV or V obesity (BMI > 50 kg/m^2^) and is considered to be a complex procedure associated with a delayed learning curve and increased concern of long-term postoperative complications [5,8,86,87]. Despite the above, literature has revealed that BPD-DS is superior in achieving sustainable short- and long-term weight loss outcomes when compared to SG and RYGB [32,88,89,90,91]. However, there remain limited long-term studies evaluating and comparing all three procedures, with the majority conducting comparative analyses of BPD-DS and RYGB.

In the randomized clinical trial by Risstad et al., patients with class IV obesity who underwent either RYGB or BPD-DS demonstrated substantial weight loss 5 years after primary MBS. However, patients undergoing BPD-DS had greater and sustained weight loss when compared to those undergoing RYGB [32]. The study reported peak weight loss outcomes at 2 years post-operatively; those findings were also consistent across other studies, demonstrating significant differences in weight loss outcomes between BPD-DS and RYGB [32,33,88,92,93,94,95].

Maroun et al. examined patients with super obesity who underwent SG, RYGB, and BPD-DS and reported that at 5 years post-operatively, BPD-DS patients experienced a significant weight reduction from baseline with a percentage total weight loss (%TWL) of 38.4% compared to the RYGB (26.3%) and SG (23.6%) cohorts. Similarly to well-published trends in RYGB and SG, the most substantial weight loss is achieved within the first 24 months following BPD-DS, after which weight loss is expected to begin to plateau. However, results are typically sustained in the long term, with very low rates of weight recurrence [95]. A study by Sethi et al. not only reported a superior short-term %EWL of 78.7% but also demonstrated a sustained long-term EWL of 67.9% at 10 years of follow-up [34], findings that are also in line with other retrospective studies demonstrating an excess weight loss of 71% maintained up to 20 years after surgery [35].

While several retrospective studies have concluded that BPD-DS results in significantly greater weight loss outcomes, this procedure does present with an increased risk of postoperative complications, such as hypoabsorptive syndromes, nutritional deficiencies, and protein-calorie malnutrition.

Hypoabsorption and nutritional complications arise with the resection and bypassing of parts of the stomach and small bowel involved in BPD-DS [36,37]. Fat-soluble vitamin deficiencies are known long-term complications following BPD-DS, which can be attributed to the intentionally shortened common channel (CC) created to allow for reduced absorption. Finno et al. also speculated that the decrease in the length of the CC results in other micronutrient deficiencies in the long term, such as zinc, calcium, iron, and copper [38], which can manifest as anemia and secondary hyperparathyroidism. A shorter CC has also been associated with an increased risk of protein malnutrition due to the limited mixing of pancreatic secretions with proteins, thereby affecting their digestion and absorption [39,96].

Sethi et al. explored nutritional outcomes in 64 BPD-DS patients and reported a 90%, 59%, 54%, 65%, and 23% rate of vitamin D, vitamin K, thiamine, zinc, and iron deficiency, respectively. Protein malnutrition and albumin deficiency were found in 40% and 15% of patients, respectively. The authors also reported that severe protein malnutrition requiring reoperation occurred in 4–5% of cases, and such surgical revision included reversal or elongation of the common channel. Of note, 1 case of severe malnutrition accounted for the long-term mortality rate 8 years after BPD-DS [34]. In addition, failed adherence to the required protein intake after surgery can further exacerbate the risk of protein-calorie malnutrition and macronutrient deficiencies. In comparison to other MBS procedures, Nelson et al. reported that the development of major nutritional complications was more frequent in BPD-DS than RYGB (4.1% vs. 2.1%), with BPD-DS patients requiring further nutritional supplementation, total parenteral nutrition, or tube feeds [33].

Lange and Königsrainer found that 69% of patients had long-term vitamin A deficiency, with the incidence of vitamins A, D, and K deficiencies increasing with time after BPD-DS. The study also reported an increased risk of thiamine deficiency in the early months following BPD-DS [37,96,97]. Additionally, Marceau et al. reported increased long-term concentrations of vitamin D following BPD-DS in patients prescribed high doses of fat-soluble vitamins [35]. Papadia et al. reported a progressive increase in the incidence of nutritional deficiencies among BPD-DS patients followed for 20–30 years, which could be explained by the lack of intestinal adaptation to the anatomical condition and absorption capability of BPD-DS over the long term. At 5 years, the most common nutritional deficiencies were protein malnutrition (10%) and anemia (8%), and at 30 years, 16% experienced protein malnutrition, 18% had anemia, 45% were diagnosed with metabolic bone disease, and 42% had micronutrient deficiency. This progressive increase in incidence reflected an increase in reoperation rates persisting until the third decade after BPD-DS [38,40].

Risstad et al. demonstrated a higher prevalence of fat-soluble vitamin deficiencies with a significant reduction in vitamins A and D levels 1 and 2 years after BPD-DS [32], which are findings consistent with other randomized [41,42] and nonrandomized studies [36,43] comparing RYGB and BPD-DS. Aasheim et al. observed that BPD-DS patients experienced lower mean vitamin A, vitamin D, and thiamine levels compared to RYGB patients. The trial also described a more common use of nutritional supplementation among BPD-DS patients (55%) compared to RYGB patients (26%) [41].

Following these findings, the study advocated for increased adoption and consideration of BPD-DS as a primary procedure in eligible patients within centers that ensure long-term follow-up and provide appropriate nutritional resources [37]. Patient education, monitoring of nutritional parameters, and additional long-term supplementation following BPD-DS are crucial. In cases where deficiencies persist despite supplementation, successful resolution of hypoabsorptive complications has been achieved through revisional bariatric surgery and lengthening of the common channel [35,98,99].

## 5. Single-Anastomosis Duodenal-Ileal Bypass with Sleeve (SADI-S)

The Single-Anastomosis Duodenal-Ileal Bypass with Sleeve (SADI-S) was originally described in 2007 [100] as a novel weight loss procedure and an alternative to the commonly performed RYGB. However, it only gained popularity in the United States after 2020 [78], and only recently has it been endorsed by the ASMBS [101].

This technique combines hypoabsorptive and restrictive effects to achieve weight loss: first, a sleeve gastrectomy is created with the standard technique but using a larger diameter calibration tube (50–54 Fr). Second, an ileal loop approximately 300 cm proximally to the ileocecal valve is marked, and the proximal duodenum is transected 2–3 cm distal to the pylorus. The marked ileal loop is finally anastomosed to the proximal end of the sectioned duodenum (Figure 3) [12,19].

Advantages of this technique include the presence of a single anastomosis and the sparing of the pyloric sphincter. SADI-S can also be employed successfully as a revisional procedure following sleeve gastrectomy, with studies showing greater %TWL (40.4% at 5 years) and higher rates of type 2 diabetes resolution (75% vs. 50% at 5 years) compared to OAGB [102]. As a primary procedure, SADI-S has been compared to both OAGB and RYGB, demonstrating higher TWL at 5 years, including in patients with very high BMIs (≥50 kg/m^2^) [103].

A study by Surve et al. found an overall complication rate of 7.8%, with no reported intraoperative complications. Most of the complications were Clavien-Dindo I or II, which led to a low rate, i.e., 0.4%, of emergency room visits within 30 days, and a readmission and reoperation rate of 1.1% each. While nausea and bleeding were the main cause of emergency department readmission, reoperation was primarily due to intra-abdominal bleeding [44]. Most short-term complications, including intra-abdominal bleeding, were managed clinically; however, surgical intervention may be necessary through either oversewing or clips after failed conservative management [104,105].

While SADI-S has demonstrated higher weight loss outcomes when compared to RYGB and SG, it also resulted in fewer long-term complications, including internal hernias, dumping syndrome, anastomotic stenosis, and anastomotic leak [44,45,106]. However, the risk of reflux remains higher when compared with RYGB and BPD-DS, which can be attributed to the increased stomach pressure and consequent bile reflux, but the overall incidence has been reported at only 1.23% according to a recent meta-analysis [46]. Commonly used in the management of reflux symptoms, proton pump inhibitors (PPIs) may further compound the risk of malnutrition associated with SADI-S. Nutritional deficiencies, such as vitamin b12 deficiency and hypomagnesemia, have been reported with long-term use of PPI therapy [107,108]. Thus, it is important to take such factors into consideration when discussing malnutrition following SADI-S.

Due to the single-loop duodenoileostomy with pylorus preservation replacing the Roux-en-Y configuration, as well as the now-standard 300-cm common limb length, a slower food passage is achieved in the small intestine, which translates to better long-term comorbidity resolution with similar outcomes when compared to RYGB [46]. As opposed to BPD-DS, SADI-S has better nutritional outcomes [47]. The most commonly found micronutrient deficiencies are folate, zinc, vitamin A, and vitamin E [48]. These deficiencies, however, are usually asymptomatic and respond well to prescribed supplementation [44,48]. Gebellí et al. demonstrated generally better blood values of iron, vitamin B12, vitamin E, and zinc in SADI-S patients compared to those in the BPD-DS group [47]. Anemia is also rare and has been reported in only 3.03% of patients [48]. Interestingly, long-term studies have reported a significant improvement in deficiency rates secondary to intestinal adaptation over time [48].

Protein malnutrition appears to be less frequent in SADI-S compared to older iterations of hypoabsorptive procedures like BPD-DS. Hu et al. described a hypoalbuminemia rate of 4.5%, with all of the affected patients not adhering to a high-protein diet [48]. Conversely, Sanchez-Pernaute et al. reported a much higher rate of 13.7% at 1 year, which can be attributed to a shorter common channel of 200–250 cm used in earlier reports [49]. The use of a 300-cm common channel in SADI-S has been associated with improved nutritional outcomes and lower rates of protein malnutrition, as described by Surve et al., whose study found a hypoalbuminemia rate of 6.2% at 1 year [50]. Steatorrhea is commonly found in the first year postoperatively but is usually managed clinically with dietary adjustments or cholestyramine [49].

Severe malnutrition necessitating reoperation is rare after SADI-S. Surve et al. reported rates ranging from 0.2% to 4.1%, with some cases linked to underlying conditions such as major depressive disorder or non-adherence to dietary recommendations. Notably, the need for revisional surgery for malnutrition in SADI-S (approximately 0.7%) is lower than in traditional BPD-DS, highlighting its safety and improved nutritional profile [44].

Overall, while SADI-S is associated with micronutrient deficiencies similar to other hypoabsorptive bariatric procedures, its design—particularly the longer common channel—contributes to better nutritional outcomes, lower rates of severe malnutrition, and improved long-term patient management.

## 6. Limitations and Future Directions

While we provide a comprehensive and scoping review of the current literature on the prevalence of malnutrition following hypoabsorptive procedures, certain limitations should be addressed. This review was conducted as a narrative review rather than a systematic one. Additionally, most studies on the topic are retrospective in nature with varying follow-up times, patient populations, and measured outcomes. This variability introduces bias, limiting the reliability and generalizability of the studies. Although systematic reviews provide more comprehensive evidence, the lack of rigorous prospective studies and randomized clinical trials (RCTs), especially on the newer techniques like OAGB and SADI, represents a critical gap in the evidence. Thus, further prospective studies and RCTs are needed to generate robust conclusions and guidelines to aid in the recognition and management of nutritional deficiencies following these procedures. Additionally, future research should also explore the impact of patient factors, such as preoperative nutritional status, as well as the role of postoperative nutritional management and medications, on the outcomes of hypoabsorption.

## 7. Conclusions

Hypoabsorptive procedures, such as OAGB, BPD-DS, and SADI-S, serve as powerful and vital choices in the surgical management of obesity. Their combined restrictive and bypass elements induce substantial and sustained weight loss, with comparable and superior outcomes compared to the more commonly performed RYGB and SG procedures, especially in patients with higher BMI (>50 kg/m^2^). The significant metabolic improvements demonstrated with these operations make them particularly valuable for patients with obesity-related medical conditions. However, these benefits must be balanced with their risk for malnutrition and nutritional deficiencies, highlighting the need for careful patient selection and long-term follow-up. As evidence on long-term outcomes continues to grow, future research should focus on optimizing these procedures and improving patient safety. Ultimately, hypoabsorptive bariatric surgeries play a vital role in the management of obesity in patients in which other procedures may fall short.

## Figures and Tables

**Table 1:** Summary of Nutritional Outcomes from Key Studies.

Bariatric Procedure	First Author	Publication Year	Type of Article	Outcomes
One-anastomosis Gastric Bypass (OAGB)	Bandlamudi, N. [15]	2023	Systematic Review	- Malnutrition outcomes after OAGB. - Severe malnutrition in 0.9% of patients, requiring in-hospital treatment and surgical revision. - 0.02% mortality.
	Carbajo, M.A. [20]	2017	Retrospective Study	- OAGB long-term outcomes. - Malnutrition in 1.1% of cohort, severe iron deficiency anemia (parenteral nutrition) in 1.25%, variable rates of vitamin B12 deficiency (2.5–21.9%) over 5 years.
	Robert, M. [21]	2019	Randomized Controlled Trial	- OAGB vs. Roux-enY gastric bypass (RYGB) safety and efficacy. - 10.8% incidence of malnutrition in OAGB group at 2 years, compared to 16.7% in RYGB group. - Significant difference in hemoglobin reduction in OAGB group (−10.3 g/L) vs. RYGB (−3.0 g/L); no difference in malnutrition, iron deficiency, or vitamin deficiencies.
	Liagre, A. [22]	2020	Retrospective Study	- OAGB with 150 cm biliopancreatic (BP) limb with 8 years of follow up. - Mild hypoalbuminemia (8%), anemia (12%), vitamin A deficiency (54%), vitamin D deficiency (33%).
	Mahawar, K.K. [23]	2018	Observational Study and Review	- Impact of BP limb length on malnutrition. - 0.6% rate of severe protein-calorie malnutrition with BP limb > 250 cm, 0.51% with BP limb > 200 cm, 0% with BP limb of 150 cm.
	Zarshenas, N. [24]	2021	Retrospective Study	- OAGB vs. RYGB outcomes after 2 years. - Higher rate of vitamin b12, folate, and homocysteine deficiencies in OAGB group compared to RYGB, with no statistical significance.
	Kessler, Y. [25]	2020	Prospective Study	- Nutritional outcomes following OAGB. - Significant reduction at 12–20 months postop in folate, vitamin D, and hemoglobin levels. - Severe anemia (<8 g/dL) in 1.2% of cohort.
	Parmar, C.D. [26]	2018	Systematic Review	- OAGB outcomes with 6 months to 12 years follow up period. - 7% rate of anemia, 0.71% rate of malnutrition.
	Jammu, G.S. [27]	2016	Prospective Study	- OAGB outcomes compared with RYGB and sleeve gastrectomy (SG). - Hypoalbuminemia in 13.1% of OAGB group, compared to 2% in RYGB and nil in SG. - Anemia in 4.9% of OAGB, 4.8% of RYGB, and 3.5% of SG group.
	Syn, N.L. [28]	2020	Prospective Study	- Micronutrient deficiencies in OAGB vs. RYGB and SG. - Steep decrease in hemoglobin levels 3 months postop in OAGB and RYGB groups.
	Shirazi, N. [29]	2022	Retrospective Study	- OAGB vs. SG nutritional outcomes. - Significant decrease in hemoglobin in both groups (greater in OAGB) with normal levels at 1 year postop. - Greater reduction in albumin levels in OAGB group (within normal range).
	Voglino, C. [30]	2021	Retrospective Study	- Mid-term outcomes of OAGB vs. RYGB. - Higher rate of vitamin A deficiency in OAGB (1.8%) vs. RYGB (0%). - Similar rates of hemoglobin reduction, vitamin D deficiency, and vitamin E deficiency. - No significant difference in malnutrition (37% in OAGB vs. 38% in RYGB had mild malnutrition; no cases of moderate or severe malnutrition).
	Cao, L. [31]	2023	Meta-Analysis	- Mineral status change after OAGB, RYGB, and SG. - Higher rates of zinc and iron deficiencies in OAGB group.
Biliopancreatic Diversion with Duodenal Switch (BPD-DS)	Risstad, H. [32]	2015	Randomized Controlled Trial	- Five-year outcomes of BPD-DS vs. RYGB in patients with BMI of 50–60 kg/m^2^. - Significantly greater reductions in vitamin A, vitamin D, and calcium levels in BPD-DS group. - 13.8% of BPD-DS group had protein-calorie malnutrition requiring hospitalization, compared to 0% in RYGB group.
	Nelson, D.W. [33]	2012	Retrospective Study	- BPD-DS vs. RYGB outcomes after 5 years. - Significantly higher incidence of any nutritional deficiencies in BPD-DS group (4.1%) vs. RYGB (2.1%).
	Sethi, M. [34]	2016	Retrospective Study	- Long-term outcomes after BPD and BPD-DS. - Micronutrient deficiencies included vitamin D (89%), vitamin K (65%), zinc (65%), anemia (57%). - Protein and albumin deficiencies in 40% and 18% of cohort, respectively.
	Marceau, P. [35]	2015	Retrospective Study	- Long-term outcomes of BPD-DS. - Nutritional deficiencies (calcium, iron, vitamin A) were present in 2% of cohort.
	Topart, P. [36]	2014	Retrospective Study	- Nutritional outcomes after BPD-DS. - At 2 years postop, >20% of cohort had vitamin A and iron deficiency and 70% had vitamin D deficiency despite supplementation regimen.
	Strain, G.W. [37]	2017	Retrospective Study	- Nutritional outcomes 9 years after BPD-DS. - Protein deficiency appeared at 3 years postop, increasing to 30% at 9 years. - 45% had zinc deficiency at 5 years. - 20% had iron and vitamin A deficiency at 9 years.
	Finno, P. [38]	2020	Retrospective Study	- Weight loss and safety outcomes in BPD-DS vs. SADI-S. - Hypoalbuminemia in 0.8% of BPD-DS patients, 0% in SADI-S. - Higher rates of vitamin A, D, and E deficiencies in BPD-DS group.
	Salame, M. [39]	2025	Retrospective Study	- Effect of common channel (CC) and Roux limb (RL) length on BPD-DS outcomes. - Overall malnutrition rate of 1.7%; higher rate (5%) in CC/RL 100/150 cm group compared to 150/150 cm (4.7%) and 125/125 cm (1%) group.
	Papadia, F.S. [40]	2024	Retrospective Study	- Nutritional outcomes of BPD-DS after 30 years. - Nutritional deficiencies were present in 60% of cohort at 20 years and 74% at 30 years, most commonly anemia and protein malnutrition.
	Aasheim, E.T. [41]	2009	Randomized Controlled Trial	- Vitamin status 1 year after BPD-DS vs. RYGB. - Lower vitamin A, vitamin D, thiamine, and hemoglobin concentrations in BPD-DS patients.
	Søvik, T.T. [42]	2011	Randomized Controlled Trial	- Outcomes 2 years after BPD-DS vs. RYGB. - Lower vitamin A and D concentrations in BPD-DS group. - 10.3% protein-calorie malnutrition rate in BPD-DS compared to 0% in RYGB group.
	Magee, C.J. [43]	2011	Retrospective Study	- Outcomes 4 years after BPD-DS. - 3.3% of cohort developed severe protein-calorie malnutrition requiring supplementary enteral feeding. - Symptomatic vitamin A deficiency in 3.3% of patients.
Single-anastomosis Duodenoileostomy with Sleeve (SADI-S)	Surve, A. [44]	2020	Retrospective Study	- Long-term outcomes after SADI-S. - Significantly lower levels of albumin, total protein, and vitamin E concentration 5 years postop; none were symptomatic.
	Pennestrì, F. [45]	2022	Retrospective Study	- Safety and efficacy outcomes of SADI-S. - 1.7% rate of malnutrition, 0.8% requiring revision to RYGB.
	Yashkov, Y. [46]	2021	Retrospective Study	- 5-year outcomes of SADI-S vs. Roux-en-Y Duodenal Switch. - Lower incidence of clinically significant protein deficiency n SADI-S (1.3%) vs. RY-DS (8.3%) group.
	Gebellí, J.P. [47]	2022	Retrospective Study	- 5-year outcomes after SADI-S vs. DS in patient with grade IV obesity. - Significantly lower levels of vitamin B1, iron, vitamin E, and zinc in DS group compared to SADI-S group.
	Hu, L. [48]	2024	Retrospective Study	- Short-term SADI-S outcomes. - 6.1% incidence of anemia, 4.5% hypoalbuminemia, 24.2% zinc deficiency, 13.6% iron deficiency, 3% magnesium deficiency, 4.5% calcium deficiency. - 45.5% vitamin A deficiency and 25.8% vitamin E deficiency.
	Sánchez-Pernaute, A. [49]	2022	Retrospective Study	- Long-term outcomes of SADI-S. - 7.3% incidence of severe protein-calorie malnutrition requiring surgical revision.
	Surve, A. [50]	2020	Retrospective Study	- 2-year outcomes after SADI-S - 8.6% of patient had significant reduction in vitamin D, 22.9% in ferritin, 17.1% in calcium concentrations at 12 months.

## Data Availability

Not applicable.

## Abbreviations

The following abbreviations are used in this manuscript:

MBS	Metabolic and bariatric surgery
SG	Sleeve gastrectomy
RYGB	Roux-en-Y gastric bypass
OAGB	One-anastomosis gastric bypass
ASMBS	American Society for Metabolic and Bariatric Surgery
BPD-DS	Biliopancreatic diversion with duodenal switch
SADI-S	Single-anastomosis duodenal switch with sleeve
BMI	Body mass index
GLP-1	Glucagon-like peptide-1
%EWL	Percentage excess weight loss
%TWL	Percentage total weight loss
CC	Common channel
